# Effects on Biomarkers in Stress Ecology Studies. Well, So What? What Now?

**DOI:** 10.3390/biology11121777

**Published:** 2022-12-08

**Authors:** Marco F. L. Lemos, Bernardo Duarte, Vanessa F. Fonseca, Sara C. Novais

**Affiliations:** 1 MARE-Marine and Environmental Sciences Centre & ARNET—Aquatic Research Network Associated Laboratory, ESTM, Polytechnic of Leiria, 2520-641 Peniche, Portugal; 2 MARE-Marine and Environmental Sciences Centre & ARNET—Aquatic Research Network Associated Laboratory, Faculty of Sciences, University of Lisbon, Campo Grande, 1749-016 Lisbon, Portugal; 3 Departamento de Biologia Vegetal, Faculty of Sciences, University of Lisbon, Campo Grande, 1749-016 Lisbon, Portugal; 4 Departamento de Biologia Animal, Faculty of Sciences, University of Lisbon, Campo Grande, 1749-016 Lisbon, Portugal

## 1. Biomarkers in Stress Ecology—From the Gene to Population

Effects assessed at higher levels of biological organization (populations and communities) are the consequence of the sum of effects on individuals, which usually result from impacts at cellular and molecular levels. Given this rationale, these lower levels of biological organization are more responsive at an early stage, making them potential resources that can be used as early warning endpoints to address environmental stress. In this way, the information concerning effects at the molecular level of biological organization (e.g., transcripts, proteins, or metabolites) allows for an early assessment of future ecosystem problems, which may eventually enable a timely intervention before the impacts become visible and irreversible. However, despite providing an early warning and a better understanding of the toxicity mechanisms, enabling the protection of biological integrity, the most significant setback is that these endpoints may fail to foresee later impacts on the environment due to the ecosystem resilience or a weak link to the effects in the following level of biological organization, making these tools simply too conservative for stakeholders’ interests. Hence, an approach targeting lower levels of biological organization will greatly benefit from addressing potential effects at higher levels. This can be achieved by establishing a link in biological organization, where the effects assessed at the lower end of biological organization are linked with the high probability of causing an effect at the other end, inducing changes in populations and communities, and eventually altering ecosystems in the future.

## 2. This Special Issue 

Within this framework, this Special Issue covers perspective articles and research papers addressing sub-individual endpoints/biomarkers in diverse environments and organisms, where biomarker effects are detected early, and the link to potential impacts in a future ecosystem is explored in six high-quality manuscripts. 

A comprehensive overview of biomarker studies in stress biology and ecology is provided by Marco Lemos [1] in a perspective article on the link between genes and the population, as well as the myriad of ecosystems and organisms in which one can use biomarkers, while providing an approach to their different applications. This publication provides the entire framework for this Special Issue, by introducing biomarkers and exploring their use, which to choose, how to communicate them and their applications and perspectives, stating a myriad of possibilities for the use of biomarkers in the field of stress biology and ecology. Several examples are given considering the paramount importance of establishing a link between sub-individual level responses and high levels of biological organization effects, which ultimately, while also describing organisms’ metabolism and physiology, validates the use of biomarkers. Although an independent study, this manuscript sets the ground for the use of a vast array of tools to be used as biomarkers in an equally vast array of taxa and for a wide selection of applications, as the other studies included in the Special Issue demonstrate.

In the study by Daniel Crespo et al. [2], biomarkers were used to address the potential of invasive bivalves (*Ruditapes philippinarum*) succeeding over their native counterparts (*R. decussatus*) in a new geographical area under a climate change scenario, thus altering the dynamics of the trade of this clam and important ecological processes. After being exposed to a heat wave setup, besides bioturbation and nutrient generation, the authors analysed oxidative stress and damage, as well as energy metabolism biomarkers. Despite no ecological level responses being detected, biomarkers point towards a lesser impact on the invasive clam under a heat wave, which may further imply improved fitness and competitive behaviour under stress. This study provides a framework where biomarkers may be used to estimate future species dynamics under global changes in an emergent topic, such as bioinvasions.

Jadilson Damasceno et al. [3] addressed mortality, behaviour, and neuromuscular, detoxification, oxidative stress, and energy metabolism biomarkers in a green crab (*Carcinus maenas)* exposed to sulfoxaflor, an insecticide, acting as a nicotinic acetylcholine receptor (nAChRs) agonist. These authors found that sufoxaflor affects the green crab’s behaviour, reduces its detoxification capacity and energy metabolism, and increases lipid peroxidation. This theoretical approach, as stated by the authors, explained the impacts of a lesser-known pesticide in these non-target crustaceans as well as helped to depict its toxicological mode of action.

Yulia Sapozhnikova et al. [4] published their paper on the sex-associated effects of noise pollution in a vertebrate, the fish stone sculpin (*Paracottus knerii*), by performing a comparative analysis of the macula sacculi and an assessment of haematological and molecular stress responses. From the addressed endpoints, exposure to noise resulted in changes in some cytometric parameters in blood, hair cell loss, and an increase in the number of functionally active mitochondria in the red blood cells of males and its decrease in females. They also found a decrease in the telomerase activity of the auditory epithelium and shorter telomeres in the brain, but only in females. Conversely, in the male gonads, an increase in telomerase activity was observed. These findings concerning sex-associated effects could possibly be associated with accelerated cellular ageing in females and lower stress-related long-term risk in males. The biomarker approach used here further corroborates the view that these tools are useful for studying the impacts of human-induced rapid environmental changes, which may be helpful for understanding and mitigating these effects through effective environmental management practices.

The following studies exemplify that biomarkers are transversal to virtually all taxa, not only in the animal kingdom, but also in algae. Zaniel Procopio et al. [5] conducted a multifactorial evaluation of the micropollutants atenolol, caffeine, carbamazepine, and ibuprofen on microalgae *Raphidocelis subcapitata* and *Chlorella vulgaris*. They evaluated cell size, photosynthetic capacity, and growth rate, and these data were then combined into a “the index effect”, a multifactorial analysis enabling the quantification and practical visualization of the pharmaceutical’s effects on these algal cultures. This study shows that environmentally relevant concentrations of the compounds affect both microalgae, being concentration and time-dependent. Additionally, these authors argue that pharmaceuticals enhance microalgae development, which may result in an imbalance of ecosystems.

Additionally, in microalgae (marine diatom *Phaeodactylum tricornutum*), Nuno Rodrigues et al. [6] used a deep learning artificial intelligence approach to tackle bio-optical data in diatoms exposed to legacy and emerging contaminants, including pesticides, cosmetics, personal and household care products, and pharmaceuticals. By merging an ecotoxicological approach with the most advanced optical high-throughput phenotyping grounded in deep learning artificial intelligence methods, it was possible to assess not only the type of contaminant to which the diatoms were exposed, but also the dose that was applied in each experimental unit. The results indicate that 2D convolutional neural networks are the best method to predict the type of contaminants to which the cultures were exposed, achieving a median accuracy of 97.65%, while Rocket is the best method at predicting which concentration the cultures were exposed, achieving a median accuracy of 100%. This study opens new perspectives for the inclusion of artificial intelligence automatic systems in biomarker interpretation and ecotoxicological classification approaches, allowing the use of large datasets with a high degree of accuracy.

## 3. The Future and Challenges of Biomarkers

The papers of this Special Issue highlight the vast potential that biomarkers have in diverse taxa and environments, especially when these are addressed early and a link to potential impacts on future ecosystems is established. This approach may potentially allow problems to be solved before impacts become visible and irreversible at higher levels of biological organization. On the other hand, by not establishing a link, biomarkers may become simply too conservative for regulatory and policy makers purposes. The latter also puts the spotlight on the biomarkers or set of biomarkers that should be chosen for a given purpose, with this decision being vital for a given application or conclusion. This decision task often requires one to opt for either a few options that may provide an incomplete overview of effects, or a more complete set that will often send the researcher into a myriad of misleading interpretations or dead ends, which today are less of a burden due to bioinformatic tools and artificial intelligence, helping to simplify and interconnect the data. Moreover, every day, more complete and comprehensive statistical tools allow for the simplification and communication of these data in multifactorial analyses, enabling a practical visualization of the results of integrating effects: Crespo et al. [2], Damasceno et al. [3], Procopio et al. [5], and Rodrigues et al. [6].

The use of biomarkers is not new and is recognized as robust and useful in several applications. However, what is preventing them from being used in environmental risk assessments and incorporated in regulatory legislation? There are limitations and challenges as stated in Lemos [1], but as this author clearly states, if “one understands the nature of such limitations and that not all biomarkers are useful for a particular need, and when a well-planned experimental design is used, biomarkers may be used as reliable and sensible tools with great explanatory potential. Its usefulness is thus unquestionable”.

The use of biomarkers in stress biology and ecology will continue to thrive, and much robust information will continue to be generated. It is up to researchers and other stakeholders to make this information worthwhile and transpose it to real setup applications, but always bearing that a link from the biomarker to high levels of biological organization will give this tool the relevance that it needs.
